# Ferroptosis-based drug delivery system as a new therapeutic opportunity for brain tumors

**DOI:** 10.3389/fonc.2023.1084289

**Published:** 2023-02-23

**Authors:** Yansheng Yao, Peng Ji, Hao Chen, Jianwen Ge, Yajing Xu, Peng Wang, Li Xu, Zhirong Yan

**Affiliations:** ^1^ Department of Endocrinology, The Affiliated Taixing People’s Hospital of Medical College, Yangzhou University, Taixing, China; ^2^ College of Pharmacy and Chemistry & Chemical Engineering, Jiangsu Provincial Key Laboratory of Chiral Pharmaceutical Chemicals Biologically Manufacturing, Taizhou University, Taizhou, China; ^3^ Department of Nursing, Liaoning Vocational College of Medicine, Shenyang, China; ^4^ Department of Anesthesiology, Fujian Maternity and Child Health Hospital, College of Clinical Medicine for Obstetrics & Gynecology and Pediatrics, Fujian Medical University, Fujian Key Laboratory of Women and Children’s Critical Diseases Research, Fujian, China

**Keywords:** ferroptosis, brain tumors, nano drug delivery systems, glioblastoma, neuroblastoma

## Abstract

The brain tumor is a kind of malignant tumor with brutal treatment, high recurrence rate, and poor prognosis, and the incidence and death rate is increasing yearly. Surgery is often used to remove the primary tumor, supplemented by radiotherapy and chemotherapy, which have highly toxic side effects. Therefore, there is an urgent need to explore new strategies, methods, and technologies that can genuinely improve the treatment of brain tumors. Ferroptosis differs from traditional apoptosis’s morphological and biochemical characteristics, and ferroptosis possesses its unique characteristics and mechanisms, opening up a new field of ferroptosis treatment for cancer. It has been found that there is a close relationship between ferroptosis and brain tumors, and a novel nano-drug delivery system based on ferroptosis has been used for the ferroptosis treatment of brain tumors with remarkable effects. This review firstly analyzes the characteristics of ferroptosis, summarizes the mechanism of its occurrence and some factors that can be involved in the regulation of ferroptosis, introduces the potential link between ferroptosis and brain tumors, and clarifies the feasibility of ferroptosis in the treatment of brain tumors. It then presents the ferroptosis nano drug delivery systems developed under different metabolic pathways for ferroptosis treatment of brain tumors. Finally, it summarizes the current problems and solutions of ferroptosis nano drugs for brain tumor treatment, aiming to provide a reference for developing ferroptosis nano drugs against brain tumors.

## 1 Introduction

Brain tumors are different types in the brain’s central nervous system (CNS) and are mainly divided into primary and secondary (1). Due to the specificity of the site of occurrence, brain tumors have become one of the essential tumors that endanger human life and health, and the disease burden is increasing year by year. In 2018, the estimated number of new cases of brain tumors worldwide was close to 300,000, accounting for about 1.6% of all new cases of malignant tumors and ranking 17th in the incidence of malignant tumors (2). Therefore, the treatment of brain tumors has been a global problem. The factors causing brain tumors are partly exogenous factors, including bad habits in life, environmental pollution, radioactive elements, etc. In contrast, endogenous factors involve hereditary genetic factors, congenital or acquired immune defects, etc (3). After the brain tumor grows unrestrainedly in the skull to a certain extent, it will compress the local tissues or nerves and increase the intracranial pressure, which will cause paralysis and mental disorder and seriously endanger the life and health of the patient. Surgery is the best treatment option for patients with advanced brain tumors, which can be supplemented with radiotherapy or chemotherapy to achieve the best treatment effect (4). However, for some advanced brain tumors, due to the extensive infiltrative growth of malignant tumor cells into the skull, it is difficult to distinguish them from normal cells, which increases the difficulty of tumor cell resection, and the higher the grade of malignant brain tumor, the higher the malignancy and the worse the prognosis (5).

The traditional cell death modalities are apoptosis, autophagy, and necrosis (6). Ferroptosis is a newly discovered mode of cell death in recent years, which differs from traditional cell death in terms of morphology, mechanism of onset, and biochemical characteristics (7). Ferroptosis is an iron-dependent, concomitant lipid peroxide accumulation mode of death (8). Many studies at this stage have shown that many malignancies, including brain tumors, exhibit sensitivity to ferroptosis (9, 10). Iron is an essential element in living organisms, especially for malignant tumor cells that require it more to maintain their vital activities such as proliferation, differentiation, and migration (11). Ferroptosis has attracted much attention in the anti-tumor field, and many ferroptosis-based drugs have been gradually applied in clinical treatment with specific effects (12, 13). However, due to the high complexity of the human brain, the high invasiveness of tumor cells, tumor heterogeneity, and the existence of the blood-brain barrier (BBB), many factors hinder the effective delivery of therapeutic drugs to tumors, resulting in insufficient drug accumulation or even acquired tumor resistance. These limitations significantly reduce the effectiveness of ferroptosis therapy (14, 15). Therefore, there is an urgent need to develop ferroptosis drug delivery systems that can efficiently cross the BBB and target tumor cells at brain lesion sites.

Booming nanomaterials offer a promising platform for the safe and efficient treatment of tumors (16). Nanomaterials can deliver drugs directly to the focal area to improve efficacy and can be transported by the bloodstream (17). Its effect is exerted in the focal area rather than the whole body, enhancing anti-tumor efficiency while reducing damage to normal body tissues (18). Nanomedicine refers to the use of nanotechnology and formulation science to carry an API (or active molecule) in a nano-sized (1-1000 nm) drug carrier, also known as a nanocapsule (19). In recent years, the successful development of nanomaterials and technologies has provided a promising platform for the safe and efficient treatment of brain tumor nano drugs (20). The properties of nanomedicines are manifested as 1) effective increase in drug solubility, 2) adequate protection of unstable drugs against premature degradation, 3) tumor targeting through passive or active targeting mechanisms, and 4) multiple drugs can be delivered by one nanocarrier to exert synergistic effects (21, 22). Ferroptosis nano drugs designed for the unique environment of brain tumors can protect the loading components, target brain tumors, cross the blood-brain barrier more efficiently, reduce the damage to normal cells, improve drug accumulation and intratumoral penetration in brain tumor tissues, reduce toxic side effects on normal tissues, etc., and show excellent application value and development prospects in brain tumor therapy (23, 24). This review systematically reviewed the ferroptosis nano drug delivery systems designed by different metabolic pathways, the current status of clinical applications, challenges, and opportunities based on the metabolic pathways of ferroptosis.

## 2 Overview of ferroptosis

### 2.1 Basic concepts and characteristics

The definition of ferroptosis was first described in 2012 when Dixon et al. studying human fibrosarcoma cells, found a significant increase in intracellular lipid reactive oxygen species (ROS) after treatment with the anti-tumor drug elastin and the concomitant appearance of cell separation and death (25). However, the number of cell deaths was reversely reduced with an iron chelator, so they speculated that the concentration of iron ions and lipid ROS influenced this mode of cell death. They formally named this mode of death ferroptosis.

Ferroptosis is a newly discovered form of programmed cell death (PCD) that distinguishes itself from traditional cell death modalities such as apoptosis, cell necrosis, and cell autophagy (26). Ferroptosis is mainly caused by the imbalance between the production and degradation of intracellular lipid reactive oxygen species. When the cellular antioxidant capacity is reduced and lipid reactive oxygen species accumulate, it can cause cellular ferroptosis (27). Ferroptosis is iron-dependent and is characterized by a lipid peroxide-aggregated cell death pattern that differs significantly from traditional cell death modalities such as apoptosis, autophagy, and necrosis at the cell morphology, biochemical characteristics, and genetic level (28, 29). Morphological aspects of ferroptosis are manifested by smaller mitochondria, increased density of mitochondrial membranes, progressive contraction of mitochondria and reduction or disappearance of cristae, breakage of the outer mitochondrial membrane, and an increase in lipid reactive oxygen radicals, which maintain the cell membrane without rupture while chromosomes are not condensed (30). The biochemical features of ferroptosis include the aggregation of iron ions, glutathione (GSH) depletion, and lipid peroxide aggregation (31). Gene-level changes in ferroptosis are manifested by the tight regulation of ferroptosis by intracellular signaling pathways, including the regulatory pathway of iron homeostasis, the RAS/Raf/MAPK pathway, and the cystine transport pathway. The immunological profile of ferroptosis is characterized by the release of proinflammatory mediators (e.g., HMGB1) by damage-associated molecular patterns (DAMPs). Susceptibility to ferroptosis is closely linked to many biological processes, including amino acid, polyunsaturated fatty acid metabolism, and the biosynthesis of GSH, phospholipids, NADPH, and coenzyme Q10 (32).

### 2.2 Mechanism of ferroptosis occurrence

#### 2.2.1 Disorders of iron metabolism

**Figure 1:**
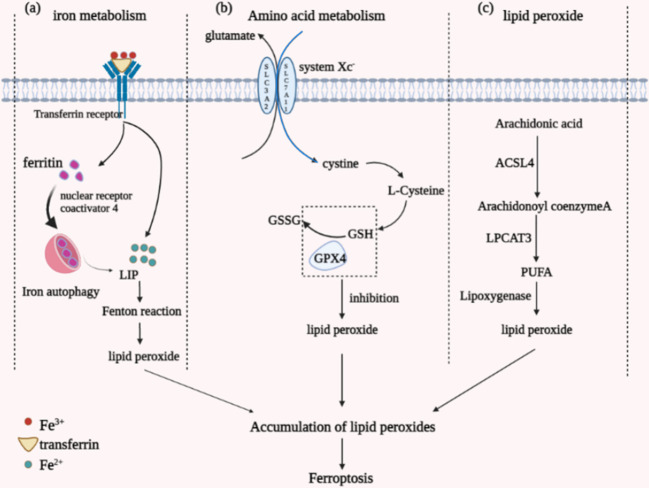
Diagram of the different mechanisms by which ferroptosis occurs. **(A)** iron metabolism; **(B)** amino acid metabolism; **(C)** lipid peroxide aggregation.

Iron, an essential trace element for the human body, is also a critical factor in the occurrence of ferroptosis (33). The main component ingested by the human body through food is Fe^3+^, which is reduced to Fe^2+^ by the action of intestinal epithelial cell reductase. After binding to transferrin, part of it is smoothly released into the cell into the unstable iron pool (LIP) with the assistance of transferrin receptors 1 and 2. In contrast, the remaining part will form ferritin. Ferritin is composed of ferritin heavy chain (FTH1) and light chain (FTL) together, which stores and regulates ferric ions (34). Iron autophagy is a process in which ferritin undergoes autophagic degradation guided by Nuclear receptor coactivator 4 (NCOA4), producing Fe^2+^ (35). Due to the high reactivity and instability of Fe^2+^, the body’s iron homeostasis is imbalanced once the Fe^2+^ in the body is overloaded. Metabolism is disturbed, making it prone to Fenton’s reaction, in which the overloaded Fe^2+^ reacts to form hydroxyl radicals, promoting the formation of lipid ROS and causing ferroptosis in cells (**Figure 1A**).

#### 2.2.2 Imbalance of amino acid metabolism

GSH, a tripeptide amino acid, is central to the metabolism of ferroptosis amino acids. It is synthesized in two steps by cysteine, glycine, and glutamate catalyzed by GSH synthase and glutamate-cysteine ligase (36). GSH has a significant effect as an antioxidant in reducing lipid peroxidation reactions and antioxidant stress by scavenging peroxides present in cells, and its regulators include glutathione peroxidase 4 (GPX4) and cystine/glutamate reverse transporter (system Xc^-^) (**Figure 1B**). GPX4 is a defensive antioxidant enzyme belonging to a selenoprotein essential for mammalian development. It can specifically catalyze the conversion of GSH to oxidized glutathione (GSSG), which reduces toxic lipid peroxides in the membrane environment to non-toxic lipid alcohols, thereby mitigating the damage caused by oxidative stress (37). Inactivating GPX4 leads to the unavailability of GSH, imbalance of amino acid metabolism *in vivo*, lipid peroxide pooling, increased damage caused by oxidation, and induction of ferroptosis.

The cystine/glutamate reverse transporter (system Xc^-^) serves as an amino acid-specific shipping protein that controls the entry and exit of amino acids into and out of cells. It is essentially a membrane Na^+^-dependent cystine/glutamate reverse transporter, mainly found in the phospholipid bilayer of biological cell membranes, formed by the glycosylated light chain subunit SLC3A2 and the non-glycosylated heavy chain subunit SLC7A11 linked by disulfide bonds, a heterodimer (38). The system Xc^-^ can transfer intracellular glutamate out of the cell while transferring extracellular cystine into the cell, which promotes the formation of GSH. By inhibiting the formation of system Xc^-^, sufficient GSH cannot be synthesized, leading to an imbalance in amino acid metabolism and ferroptosis.

In addition to the above inhibition of GSH by interfering with GPX4 and system Xc^-^, some specific compounds can be used to inhibit GSH. e.g., buthionine imine (BSO), a small molecule inhibitor that targets and inhibits GSH during GSH synthesis. APR-246 (PRIMA-1) is a toxic compound that binds GSH and depletes intracellular GSH content (39). Thus, by interfering with GPX4 expression, inhibiting system Xc^-^, or directly acting on GSH to deplete or under-synthesize it, it can imbalance amino acid metabolism *in vivo*, contributing to the accumulation of lipid ROS *in vivo* and causing ferroptosis.

#### 2.2.3 Lipid peroxide aggregation

The central aspect of ferroptosis is the iron-dependent dysregulation of lipid oxidation metabolism, and polyunsaturated fatty acids (PUFAs) are vital substances in the accumulation of lipid peroxides in ferroptosis (40). Under normal conditions, PUFAs are essential substrates for lipid peroxidation reactions and contain diallyl hydrogen atoms. PUFAs are the most fragile of lipids, and their structurally unsaturated double bonds, ester bonds between glycerol and fatty acids, are highly susceptible to lipid peroxidation by enzymes and free radicals (ROS). The process of accumulation of peroxides through the action of PUFAs consists of two main parts: the Fenton reaction and the enzymatic reaction pathway. The Fenton reaction, which is the formation of an unstable iron pool by the high activity of Fe^2+^ in the cell, generates a large amount of free radical material that can separate the hydrogen atoms in the diallyl carbon through the Fenton reaction (41), allowing the accumulation of large amounts of peroxides (**Figure 1C**).

The enzymatic process involved in the reaction is mainly lipoxygenase but also requires the participation of acetyl coenzyme A synthase long-chain family 4 (ACSL4) and lysophosphatidyl choline acyltransferase 3 (LPCAT3), which is associated with lipid remodelling (42). The reaction uses free PUFAs arachidonic acid as the primary phospholipid substrate, which is finally oxidized by lipoxygenase to form lipid peroxides after two-step esterification by ACSL4 and LPCAT3 (43). When the synthesis of the above three enzymes is inhibited, the oxidation of PUFAs to form lipid peroxides is also affected. On the contrary, excessive activation of the three enzymes or exogenous supplementation of PUFAs leads to increased oxidation, accumulation of lipid peroxides, and massive catabolism to produce toxic aldehydes such as malondialdehyde (Malondialdehyde) or 4-hydroxy-2-nonenal (4-Hydroxynonenah’yl, 4-HNE) combined with a continuous intracellular oxidation reaction that renders the organism essential proteins inactive, which triggers ferroptosis (44).

### 2.3 Mechanisms regulating ferroptosis

According to the above preliminary summary and analysis of the mechanism of ferroptosis, the trigger of ferroptosis mainly involves the disorder of iron metabolism due to the imbalance of iron ion concentration, the disorder of amino acid metabolism due to the inactivation and depletion of GSH, and the lipid peroxidation aggregation driven by polyunsaturated fatty acids. Therefore, various compounds, genes, and pathways associated with iron metabolism, amino acid metabolism, and lipid metabolism can be involved in the regulation of ferroptosis, and active regulation can trigger ferroptosis and destroy tumor cells (**Table 1**). Some of the potential regulatory mechanisms are described below.

**Table 1:** Major regulatory mechanisms of ferroptosis.

Types of regulatory pathways	Regulatory factors	References
Iron metabolism pathway	NFS1	(45)
	Heme oxygenase-1	(46)
	SLC40A1	(46)
	PCBPs	(47)
GPX4 pathway	SMG9	(48)
	CREB	(49)
	ZEB1	(50)
	RSL3	(51)
system Xc^-^ pathway	ATF3	(52)
	INFγ	(53)
	Erastin	(54)
	p53	(55)
Lipid peroxide pathway	Vitamin E	(56)
	Carbon-deuterium bond	(56)
	MOFs	(57)
Other pathways	VDAC	(58)
	CL	(59)
	FtMt	(60)

#### 2.3.1 Iron metabolism

Dixon et al. found a reversal in the number of cell deaths after adding iron chelators during ferroptosis studies, suggesting the possibility of iron metabolism on the regulation of ferroptosis. Later, Gao et al. found the effect of ferritin carrier transferrin (TF) on ferroptosis, further confirming the critical role of iron metabolic processes on ferroptosis (61). The iron metabolic process involves iron ions’ storage, transport, export, and degradation processes and contains various proteins and genes. Transferrin and transferrin receptor (TFRC) are essential regulators of iron ion transport. Cysteine desulfurase (NFS1), an iron-sulfur cluster biosynthetic enzyme, decreases ferritin expression and stimulates transferrin expression, thereby increasing the risk of ferroptosis in tumor cells (45). Heme is a Fe^2+^-containing protein catalyzed by heme oxygenase-1 to produce Fe^2+^. The Fe^2+^ generated from the degradation of large amounts of heme increases the level of iron ions in the intracellular unstable iron pool, inducing ferroptosis. Iron ions are transported into the cell by transferrin and stored in the unstable iron pool or ferritin. SLC40A1 is an iron ion transport carrier mainly responsible for the export of Fe^2+^. SLC40A1 can be regulated by heparin antimicrobial peptide, which can degrade SLC40A1 in the lysosome to reduce iron export. The accumulation of large amounts of iron ions in tumor cells causes ion metabolism to be disturbed, triggering ferroptosis (46). Polycomb binding proteins (PCBPs), an iron molecular chaperone, can participate in iron metabolic processes, on the one hand interacting with ferritin to promote the oxidation of Fe^2+^ to Fe^3+^ for storage, and on the other hand, being essential regulators of the unstable iron pool, regulating the storage and export utilization of iron ions within the iron pool (47). For example, Fe^2+^ was used to regulate the activity of cofactor iron-containing enzymes. The occurrence of ferroptosis can be controlled by regulating polycomb binding protein expression.

#### 2.3.2 GPX4

GPX4 reduces toxic lipid peroxides and is an essential effector of ferroptosis. By regulating the expression of GPX4, the accumulation of peroxides in tumor cells can be controlled, and ferroptosis can be regulated. Han et al. demonstrated the interaction between SMG9 and GPX4 after a small-scale screening of RNAi, i.e., SMG9 is a GPX4 binding protein that promotes the degradation of GPX4 protein, thereby inhibiting GPX4 activity (48). Knockdown or depletion of SMG9 content *in vivo* significantly increased GPX4 protein content and enhanced activity. CREB is a ubiquitous transcription factor that inhibits lipid peroxidation and prevents ferroptosis by binding to the promoter region of GPX4 and stimulating cell viability. Moreover, binding the protein P300 (EP300) to CREB exerts even stronger facilitation (49). ZEB1, a transcription factor, acts oppositely to CREB by repressing GPX4. ZEB1 inhibits GPX4 promoter transcriptional activity by binding to the GPX4 promoter region motif and decreases GPX4 expression (50). The advent of nano-drug vectors has also opened up more possibilities for GPX4 regulation. For example, RSL3 was originally a ferroptosis inducer that targets GPX4 and reduces GPX4 activity. Amphiphilic polymeric micelles linked to nitroimidazole-coupled peptides *via* an azobenzene linker were used to load RSL3, enabling the rapid and precise release of RSL3, which was able to significantly reduce GPX4 expression, with a twofold increase in anti-tumor efficiency compared to RSL3 (51).

#### 2.3.3 System Xc^-^

GSH prevents the accumulation of lipid peroxides and resists oxidative stress, while system Xc^-^ plays a vital role in synthesizing GSH, which becomes an essential regulator of ferroptosis. Therefore, the regulation of ferroptosis can be achieved by regulating system Xc^-^ levels. ATF3 is a transcription factor, and SLC7A11 is a critical component of system Xc^-^. ATF3 binds tightly with the promoter of SLC7A11 to regulate the transcription of SLC7A11 to achieve the repression of system Xc^-^ and reduce the synthesis of GSH. In addition, ATF3 has a facilitative effect on the reduction of GPX4 activity-induced ferroptosis by RSL because ATF3 enhances the sensitivity of RSL3 to act on GPX4, and the activity is more easily reduced (52). The glycosylation protein INFγ can target system Xc^-^ and reduce the expression levels of mRNA and protein of both SLC7A11 and SLC3A2 subunits, resulting in reduced system Xc^-^ activity and inhibition of GSH synthesis. Also, INFγ can be used with ferroptosis inducers such as RSL3 to reduce system Xc^-^ activity even more effectively, cells are blocked from G1 to S cycle, and cells die (53). Erastin is a typical ferroptosis inducer. It also achieves its effect mainly by inhibiting system Xc^-^. Still, it was also found to be counterproductive if activation of system Xc^-^ subunit SLC7A11 expression could attenuate the effect of Erastin (54). Co-loading Erastin and rapamycin constructed the nano-programmed drug delivery system through the nano-emulsification method; Erastin acts on system Xc^-^ and rapamycin acts on GPX4, which can synergistically reduce GSH synthesis and utilization and accumulation of lipid peroxides, resulting in ferroptosis of tumor cells (54). p53 is a tumor suppressor in cell proliferation, apoptosis, and other metabolic processes (62). It was also found to decrease the expression of SLC7A11 and thus inhibit system Xc^-^, increasing the susceptibility of tumor cells to ferroptosis. In addition, Erastin can upregulate the expression of p53, which further enhances the inhibitory effect and reduces the synthesis of GSH, improving the induction of ferroptosis (55).

#### 2.3.4 Lipid peroxide regulation

The accumulation of lipid peroxides is a significant cause and driver of ferroptosis. Inhibition of lipid peroxide formation can effectively stop ferroptosis from occurring, and common ways of inhibition include preventing peroxide formation and scavenging already-formed peroxides. Vitamin E is a relatively ideal antioxidant to provide electrons to peroxyl radicals, scavenging the free radicals attacking PUFAs and terminating the formation of peroxides. Another method is to label the part of PUFAs susceptible to oxidation on the diallyl with carbon-deuterium bonds because deuterium atoms are not easily replaced by oxidation. This method applies to both Fenton and enzymatic reaction pathways and has a wide range of applications to inhibit lipid peroxide formation effectively (56). Metal-organic backbones (MOFs) are crystalline materials with a periodic network structure formed by molecular self-assembly of metal ions or ionic clusters with organic ligands. Hao et al. devised a strategy for peroxisome accumulation based on catalytic MOFs for ferroptosis therapy. Bimetallic MOFs were synthesized using iron porphyrins as linkers and copper ions as metal nodes. In the tumor microenvironment, the exfoliated MOFs acted as inducers of the Fenton reaction, generating a large number of hydroxyl radicals to accumulate lipid peroxide, effectively inhibiting tumor growth in living mice and providing a new opportunity to treat tumors insensitive to apoptosis (57).

#### 2.3.5 Other regulation methods

The study of ferroptosis found that some mitochondria-related pathways also have an essential role in regulating ferroptosis. Voltage-dependent anion channel (VDAC), a mitochondrial pore protein, has a role in promoting ferroptosis. Under normal conditions, mitochondria exchange standard ions and molecules with the cytoplasmic matrix through the VDAC. When a globin microtubulin inhibits this process on the VDAC, mitochondrial metabolism is affected, leading to hyperpolarization and massive production of lipid peroxides (58). In addition, some ferroptosis inducers also affect VDAC, such as the increased permeability of the outer mitochondrial membrane, the opening of membrane ion channels, and the imbalance of intracellular homeostasis after the action of Erastin on VDAC, leading to dysfunctional mitochondrial metabolism and oxidation, increased ROS production, and enhanced lipid peroxidation, which in turn cause the development of cellular iron necrosis (63, 64). Cardiolipin (CL) is part of phospholipids in mitochondria, which has a promotional effect on ferroptosis, and it can form a complex with cytochrome C (59). Cytochrome C plays a role in the mitochondrial respiratory chain to transfer electrons, which will be destroyed when CL is peroxidized, and cytochrome C will be released to participate in the respiratory chain as an electron carrier, producing large amounts of lipid peroxides and contributing to ferroptosis in tumor cells. Mitochondrial ferritin (FtMt) is an iron storage protein found in mitochondria and can regulate iron metabolism. Wang et al. found that when FtMt was overexpressed, it had an inhibitory effect on Erastin-induced lipid peroxide production and iron ion level in the iron pool, and reasonable regulation of FtMt expression level could promote ferroptosis in tumor cells (60).

## 3 Brain tumors and ferroptosis

The two central elements that induce ferroptosis in tumor cells have been identified through the study of ferroptosis are the accumulation of iron levels or lipid peroxides within the tumor cells (65). Ferroptosis plays a vital role in anticancer research. The ferroptosis mechanism has been applied to kill relevant tumor cells (e.g., breast, gastric, and lung cancer). Recent studies have also highlighted the importance of ferroptosis in brain tumors. This article describes the progress that has been made in the means of ferroptosis in brain tumors.

### 3.1 Glioma

Glioma is an intracranial tumor that originates from glial cells of the nervous system and has a high incidence among brain tumors. It is a malignant tumor that is aggressive, drug-resistant, and has a poor prognosis (66). Traditional cancer treatment strategies (such as radiotherapy and chemotherapy) mainly target relevant genes and proteins that can induce apoptosis, e.g. Caspase-3 is one of the targets of cancer therapy, and activation of Caspase-3 plays a role in inhibiting tumor cells, but cancer cells gradually become resistant to apoptosis, which makes it difficult for traditional treatment regimens to be efficient. Ferroptosis may become an excellent alternative to solve this bottleneck. Ferroptosis as an ideal strategy for glioma treatment has the following main features: (1) glioma exhibits cell necrosis, and mesangial cells play an important role in this process. Mesangial cells accumulate on tumor cells in the early stage of glioma and activated mature mesangial cells release characteristic particles to induce lipid peroxidation in tumor cells and promote ROS production, leading to ferroptosis of tumor cells (67). It was found that gliomas have a strong capacity for lipid synthesis, and the high content of PUFAs in gliomas compared to normal cells contributes to the induction of ferroptosis in gliomas through positive regulatory mechanisms, such as increased expression of lipoxygenase or ACSL4 (68). (2) Glioma can alter the expression capacity of enzymes and proteins related to iron metabolism and accumulate iron content in tumor cells to maintain normal proliferation and metastasis processes (69). Glioma stem cells in gliomas can proliferate indefinitely and require large amounts of nutrients and metal elements for indefinite proliferation, so they also exhibit a high demand for iron. Transferrin receptors are overexpressed on glioma stem cells, and extracellular transferrin carrying large amounts of iron, which is generally mediated into cells through transferrin receptors, can overcome the standard blood-brain barrier mechanism, causing glioma stem cells to take up more iron from extracellular sources and disrupting iron metabolism in the brain. Therefore, reducing intracellular iron content by inhibiting iron uptake by glioma stem cells is a potential anticancer strategy (70). Zhang et al. found that coatomer protein complex subunit zeta 1 (COPZ1) was not only associated with increased tumor grade and poor prognosis in glioma patients but was also strongly associated with ferroptosis. Inhibition of COPZ1 expression induces ferritin phagocytosis and activates ferroptosis. elevated Fe^2+^ levels trigger the Fenton reaction, which promotes ROS production and leads to ferroptosis (71). (3) It was found that the expression of GPX4 was significantly higher in glioma tissues than in normal brain tissues. The expression of GPX4 was progressively enhanced with increasing WHO glioma grading (72). Induction of GPX4 inactivation increases intracellular lipid peroxidation leading to ferroptosis. Therefore, an effective treatment strategy is an induction of GPX4 inactivation by GSH depletion or GPX4 inhibitors (e.g., RSL3). It was also found that system Xc^-^ could assist in transporting glutamate and cystine inside and outside the cell, maintaining amino acid homeostasis, promoting GSH formation, and reducing the risk of ferroptosis. In glioma cells, when the availability of intracellular glucose decreases, glioma cells exhibit a high dependence on glutamine, and by inhibiting the formation of system Xc^-^, GSH cannot be adequately synthesized and amino acid metabolism is imbalanced, leading to ferroptosis can be achieved in the treatment of glioma. Lyuzosulfapyridine is an oral anti-inflammatory drug with an inhibitory effect on system Xc^-^, and it has been clinically used in treating glioma patients. However, there is a lack of safety, and is prone to potential neurological risks in patients with malignant glioma. Temozolomide is an alkylating anti-tumor agent that can cross the blood-cerebrospinal fluid barrier and is clinically used in the first-line treatment of brain tumors. However, some patients with glioma have a natural resistance to temozolomide and are also susceptible to temozolomide resistance during chemotherapy. Glioma cells become resistant to temozolomide by enhancing the expression of system Xc^-^ (73). Combining radiotherapy, immunotherapy, and chemotherapy with temozolomide in postoperative patients with high-grade glioma can improve efficacy, reduce drug resistance, reduce immunosuppression, improve patients’ quality of life, and prolong overall survival time. System Xc^-^ in ferroptosis will continue to be a major potential target for current and future regimens such as radiotherapy and chemotherapy. Through system Xc^-^, ferroptosis plays an important role in modulating the response of Glioma to radiotherapy, immunotherapy, and TMZ, addressing the problem of poor safety and low utilization of traditional therapeutic agents that are not available, Ferroptosis offers additional advantages. Both artemisinin and its derivatives (e.g., dihydroartemisinin and artesunate), the active ingredients extracted from the Chinese medicine Artemisia annua, can induce ferroptosis in tumor cells by enhancing heme oxygenase-1 expression and intracellular pools of unstable iron (74). Dihydroartemisinin can act as an inhibitor of GPX4 and system Xc-, increasing the accumulation of lipid peroxides in glioblastoma cells and inducing ferroptosis (75). However, its clinical application is limited because of its deficiencies, such as poor stability and poor solubility in water. The emergence of nano drug delivery carriers provides direction for anti-glioblastoma of dihydroartemisinin, such as polymeric nanoparticles and inorganic nanoparticles can be loaded with dihydroartemisinin, which improves its solubility, biocompatibility, and targeting in water and provides a new technology for ferroptosis treatment of brain tumors (74).

### 3.2 Neuroblastoma

Neuroblastoma (NB) is the most common extracranial tumor in children and the most common tumor in infants and children and is also known as the king of childhood cancers (76). Neuroblastoma is clinically widely heterogeneous, mainly exhibiting malignant tumor features such as high metastasis and susceptibility to recurrence. At the same time, a few can regress to benign tumors without treatment or even disappear entirely (77). It has been found that neural tumor cells evade ferroptosis through dopamine produced in the brain. Dopamine drives overexpression of ferritin, which redistributes iron ions that should otherwise enter the unstable iron pool of the cell into the mitochondria, resulting in a reduction of intracellular lipid ROS produced by the Fenton reaction and inhibiting ferroptosis (60). The neuroblastoma oncogenic transcription factor MYCN can also escape the risk of ferroptosis by promoting the expression of system Xc^-^ and some system Xc^-^ inhibitors (ATF3, INFγ) might increase the chance of ferroptosis in tumor cells by reducing the effect of MYCN (78). Neuroblastoma is a typical MYC-driven cancer, and patients with neuroblastoma usually present with massive amplification of the N-MYC gene (MYCN), which leads to uncontrolled cancer cells. MYCN-amplified neuroblastoma is highly cysteine-dependent and sensitive to ferroptosis. Hamed et al. performed single amino acid deprivation assays on MYCN-high-expressing neuroblastoma cells and MYCN-low-expressing neuroblastoma cells. They found that MYCN-high-expressing neuroblastoma cells were strongly dependent on cysteine, an amino acid, and that deprivation of cysteine resulted in massive death of MYCN-high-expressing cancer cells (79). This study demonstrates that when cysteine intake is restricted, cysteine is heavily used for protein synthesis, which triggers ferroptosis and can significantly inhibit terminal neuroblastoma, suggesting that the high dependence of MYCN-driven brain tumor cells on cysteine is a novel therapeutic avenue that can be exploited to induce ferroptosis in cancer cells.

### 3.3 Meningioma

Meningioma is the most common central nervous system tumor, accounting for approximately one-third of all primary brain tumors. It mainly affects the elderly, with an increased incidence over 65 years of age, more in women than men, and less frequently in children. It usually follows a benign course with a pretty good outcome, and surgery and/or radiation therapy remain the standard of care (80, 81). According to the 2016 World Health Organization classification (4th edition), meningiomas are classified into three histological grades. The prognosis remains excellent for grade I meningiomas, with an overall 10-year survival rate greater than 90%. However, while most meningiomas, especially grade I meningiomas, can be cured by surgery alone, they become clinically challenging for grade II and III recurrent meningiomas because there are no clear standard treatment options after re-excision or re-radiation. Grade III meningiomas have an inferior prognosis, with an overall 10-year survival rate of 33%. Many chemotherapeutic agents and hormonal therapies have been tried with only modest benefits (82). NF2 is a mutated gene in brain tumors, and deletion of NF2 predisposes meningiomas to ferroptosis, and E-cadherin is negatively associated with ferroptosis. The transcription factor MEF2C positively regulates the transcription of all their genes. Activation of MEF2C promotes the expression of NF2 and E-cadherin in meningiomas and causes ferroptosis in tumor cells.MEF2C may be a potential therapeutic target for ferroptosis in meningiomas (83).

## 4 Nanodelivery system mediating ferroptosis in brain tumors

**Figure 2:**
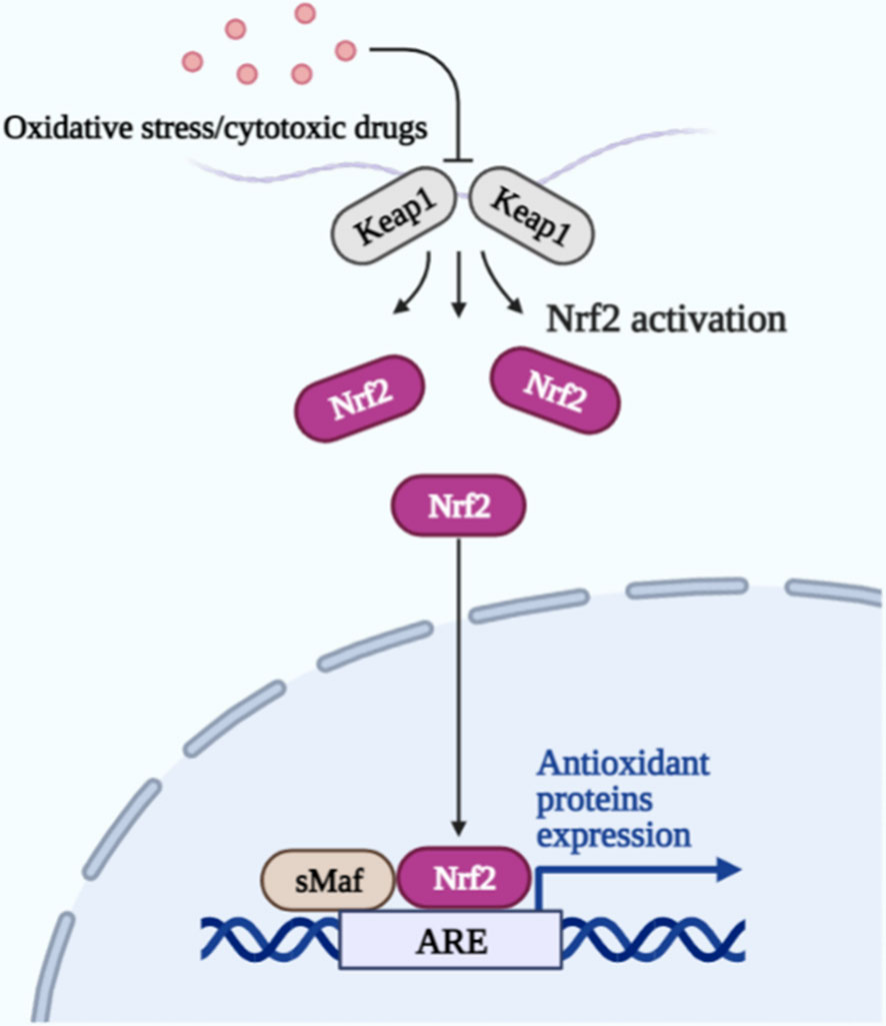
Upon oxidative stress or cytotoxic drug stimulation, Keap1 is separated from Nrf2, and Nrf2 is activated. Together with the sMaf protein in the nucleus, it binds to the antioxidant response element (ARE) and drives the expression of antioxidant protein genes to protect cells.

Some protective mechanisms exist within the brain cells to inhibit the occurrence of ferroptosis, Nrf2 is a stress-induced transcription factor that can be involved in iron metabolic processes, and GSH synthesis and metabolism-related enzymes are also under its control (84). Nrf2 can also bind to Kelch-like ECH-associated protein 1 (Keap1) and be present in the cell in an inactivated state; keap1 is the control Nrf2 produces a switch for its action. When cells encounter oxidative stress or cytotoxic drugs, the two become separated, and Nrf2 is activated. Its activation promotes iron storage, limiting ROS production and increasing the antioxidant capacity of tumor cells, making them less susceptible to ferroptosis (**Figure 2**). In addition, the blood-brain barrier (BBB) formed by the tight junctions between capillary endothelial cells in the brain, surrounded by a layer of stellate cells outside the basement membrane, prevents almost all drugs from entering the brain lesion site to exerting therapeutic effects, which is the main obstacle facing the development of drugs for the treatment of brain tumors (85). Therefore, there is an urgent need to develop drug delivery systems that can efficiently cross the BBB and target tumor cells at the focal site.

Most of the clinically used chemotherapeutic agents are apoptosis-inducing drugs, such as DOX, cisplatin, and paclitaxel. Due to their prolonged use, problems such as drug resistance, metastasis, relapse, and adverse effects have emerged (86, 87). In recent years, researchers around brain tumors have worked to develop antitumor drugs with low toxicity, high efficiency, low side effects, and high bioavailability. For example, Kar et al. found that Concanavalin A (Con A), a carbohydrate-binding protein of the cohesin family, is an ideal therapeutic agent for gliomas, and high doses of Con A inhibit the growth of glioma by disrupting the thiol/disulfide balance of tumor cells and causing oxidative stress, as well as inducing inflammatory factors and programmed apoptosis (88). In addition, Hacioglu et al. found an inhibitory effect of capsaicin on glioma (89). Kar et al. found an inhibitory effect of the trace element boron on glioma (90). Capsaicin and boron can interfere with the signaling pathways of the regulatory factors ACSL4 and GPX4 and induce ferroptosis. Although all three newly discovered chemotherapeutic agents mentioned above have the potential to treat glioma, they are all dose-dependent and require a certain precise range of dose concentrations to achieve a better inhibitory effect, such as high doses of Con A (250 and 500 µg/ml) to achieve the effect; the inhibitory effect of boron on glioma tumor cells is proportional to its dose concentration. The inhibitory effect of capsaicin on glioma started only when the concentration exceeded 50 µM. It is not difficult to find that the search for the optimal dose will inevitably prolong the drug development process, and dose dependence may become an important factor limiting the better anti-cancer effect of these chemotherapeutic drugs. In addition, the potentially toxic effects of these chemotherapeutic drugs need to be further studied, for example, the toxicity of capsaicin to normal cells is unknown, and the toxicity to organs also needs to be further studied. Therefore, there is still a long way to go before the actual clinical application.

Compared with ordinary chemotherapeutic drugs, high-end formulation nanomedicines have greater clinical application value and advantages. Specifically, (1) nanomedicines modified by targeting groups can achieve targeted drug delivery, which can reduce the dose of drugs and does not require high doses to bring the desired therapeutic effect and reduce its side effects as some chemotherapeutic drugs (e.g. Con A) do. (2) Chemotherapeutic drugs have short *in vivo* half-life and low bioavailability, which require frequent dosing and long treatment cycles for patients. Nanomedicine can solve this problem, and nanocarriers can extend the elimination half-life of drugs, increase the effective blood concentration-time, reduce the frequency of drug administration, and reduce the pain caused by treatment. (3) Nanomedicines can efficiently cross the BBB and specifically target the drug delivery system of tumor cells at the focal site, dramatically increasing their efficacy (85). (4) Nanodrugs can enter capillaries through blood circulation and also cross the endothelial cell gap to achieve targeted drug delivery and improve the bioavailability of drugs. Chemotherapeutic drugs kill tumor cells and normal tissue cells, especially the cells of blood and lymphatic tissues, which are growing vigorously in the human body and have strong killing power, which is harmful and the bioavailability of drugs is low.

Nanotherapies in the context of ferroptosis have gradually become a hot research topic. As the research on ferroptosis modulators and nanomedicine technology has steadily advanced, people have started to combine some ferroptosis modulating inducers with nanomedicine technology to construct novel ferroptosis nanomedicine delivery systems for brain tumor treatment, which not only substantially improves the targeting of the drug and makes it easier to cross the blood-brain barrier but also reduces the adverse effects and improves the bioavailability of the drug *in vivo*. Compared to other types of anticancer nano drugs, ferroptosis-based nano drugs have excellent physicochemical properties such as superparamagnetic properties, good biocompatibility, and low-cost advantages. The combination of multiple therapies is more advantageous than single therapeutic strategies in clinical cancer treatment, such as efficient synergistic therapeutic effects and reduction of toxic side effects by a single dose. Iron-dead nano drugs also have more combination therapeutic modalities than other types of nano drugs, such as magnetic iron-based nanotherapeutics that simultaneously enable the precise diagnosis of tumors through magnetic resonance imaging (MRI) (91). It can characterize different tissues of the same density and chemical structures of the same tissue by imaging display. This facilitates the differentiation of gray matter from white matter in the brain and has great superiority in the early diagnostic effect of brain tumors. The application of ferroptosis nano delivery lines based on different metabolic pathways in brain tumors is summarized in the following section (**Table 2**).

**Table 2:** Ferroptosis nano-delivery system based on different metabolic pathways.

Metabolic pathways	Nanomedicine	Type of study	Model used	Applications	Reference
Iron metabolism	FeGd-HN@LF/RGD_2_	*In vivo* *In vitro*	U-87MG	GBM	(92)
	GOD-Fe_3_O_4_@DMSNs	*In vivo* *In vitro*	U-87MG	GBM	(93)
	cRGD/Pt + DOX@GFNPs	*In vivo* *In vitro*	U-87MG	GBM	(94)
	MIONzyme-GOx	*In vitro*	U-87MG,U-118MG	GBM	(95)
Amino acid metabolism	FA/Pt-si-GPX4@IONPs	*In vivo* *In vitro*	U-87MG,P3#GBM and NHAs	GBM	(96)
	WA	*In vivo* *In vitro*	IMR-32 and SK-N-SH	NB	(97)
	35GB	*In vitro*	U-87MG	GBM	(98)
	MNP@BQR@ANG-EXO-siGPX4	*In vivo* *In vitro*	Ln229	GBM	(66)
Other	IO-LAHP NPs	*In vivo* *In vitro*	U-87MG	GBM	(99)
	IONP@PTX	*In vivo* *In vitro*	U251 and HMC3	GBM	(100)

### 4.1 Iron metabolism strategy

Ferroptosis is iron-dependent cell death, and iron metabolism is pivotal in the overall ferroptosis process. Iron metabolism involves numerous reactive processes of iron ions, during which many regulatory proteins and genes (ferritin, transferrin) are involved. Its highly complex process, mediated by any pathway, affects the intracellular iron ion content and impacts iron metabolism. Many ferroptosis nano drug delivery systems are designed based on iron metabolism pathways. They are primarily iron-based nanomaterials, which act on specific reaction sites of tumor cells through their high iron content and participate in the Fenton reaction process, increasing the formation of lipid ROS and causing ferroptosis of tumor cells.

Shen et al. designed a FeGd-HN@LF/RGD_2_ nanoparticle, a hybridized nanoparticle formed by coupling lactoferrin, RGD dimer, and cisplatin loaded with Fe_3_O_4_/Gd_2_O_3_. The nanoparticles can release Fe^3+^ and Fe^2+^ to participate in the Fenton reaction and promote lipid ROS production in brain tumor cells. At the same time, the cisplatin fraction can stimulate the production of hydrogen peroxide (H_2_O_2_), another substrate of the Fenton reaction, jointly accelerating the Fenton reaction and inducing ferroptosis in brain tumor cells (92). GOD-Fe_3_O_4_@DMSNs are ferroptosis nanocatalysts with excellent biodegradability and compatibility made from natural glucose oxidase (GOD) and ultra-small Fe_3_O_4_ nanoparticles integrated into large pore size and degradable dendritic silica nanoparticles. This ferroptosis nanocatalyst was found to release natural glucose oxidase *in vivo*, consume glucose from brain tumor cells, and produce hydrogen peroxide to promote the Fenton reaction, which can trigger ferroptosis and effectively inhibit the activity of glioma cells, providing a promising ferroptosis treatment strategy for glioma patients (93). Zhang et al. extracted gallic acid from gallus compounded it with Fe^2+^ into a nanocarrier (GFNP), and co-loaded the precursor drug inert Pt and chemotherapeutic drug adriamycin to construct cRGD/Pt + DOX@GFNPs nanoformulations. *In vivo* evaluation showed that GFNPs could induce ferroptosis by generating Fe^2+^ to promote the Fenton reaction and produce lipid ROS. In addition, Pt and adriamycin could induce apoptosis in brain tumor cells, ultimately obtaining ferroptosis synergistic with apoptosis to inhibit glioma efficiently (94). Alexandra et al. prepared a hybrid nano preparation (MIONzyme-GOx) based on nano preparation by coupling a natural enzyme (GOx) with an iron oxide-based nano preparation (MIONzyme) and wrapping it with a biocompatible carboxymethylcellulose as a shell was prepared (95). The results of *in vitro* experiments on brain tumor cells showed that the GOx released from this preparation could generate H_2_O_2_ with glucose, which was then catalyzed by iron oxide nanoparticles to promote the Fenton reaction and generate lipid ROS, which further induced ferroptosis and successfully inhibited brain tumor cell proliferation.

### 4.2 Amino acid metabolic strategy

Amino acid metabolism involves the antioxidant GSH, which effectively avoids intracellular peroxide production. GPX4 and system Xc^-^, which are involved in the utilization and synthesis of GSH, respectively, and also play essential roles in the stabilization of amino acid metabolism, have also been described previously, both belonging to the critical regulators of ferroptosis. This paragraph reviews nano drugs that inhibit system Xc^-^ or the GPX4 pathway to interfere with normal amino acid metabolism and thus induce ferroptosis.

Zhang et al. constructed a nano drug (FA/Pt-si-GPX4@IONPs) based on the specific pathophysiological characteristics of glioblastoma (GBM), which is based on porous iron oxide nanoparticles (IONPs) that carry cisplatin (Pt) and small interfering RNA (si-GPX4) inside, and the surface of the drug-carrying IONPs is modified with Folic acid, which can bind to the highly expressed folate receptor on the surface of glioma (96). *In vitro*, cytological experiments showed that FA/Pt-si-GPX4@IONPs produced significant killing effects on GBM cells but not on normal human astrocytes (NHA). During the intracellular degradation of nano drugs, IONPs significantly increased iron and ferrous ions (Fe^2+^ and Fe^3+^) levels in GBM cells (96). They generated strongly cytotoxic hydroxyl radicals by the Fenton reaction between them and H_2_O_2_, which oxidized unsaturated fatty acids and triggered ferroptosis. At the same time, the piggybacked si-GPX4 inhibited the expression of GPX4, synergistically improving the effect of induced ferroptosis. The therapeutic approach achieved significant results both *in vitro* and *in vivo*, and FA/Pt-si-GPX4@IONPs nano drugs are expected to be applied in treating GBM. Hassannia et al. identified withaferin A (WA), a natural ferritin inducer in neuroblastoma, to inhibit neuroblastoma cell appreciation as well as inhibit the growth and recurrence of murine neuroblastoma heterogeneous tumors by inhibiting GPX4 or targeting Keap1 to increase the unstable iron pool, which in turn suggested a new therapeutic strategy by iron induction to kill cancer cells effectively. The use of multifunctional nanocarriers with targeting, degradability, and pH sensitivity to wrap WA for the preparation of nanomedicines can solve the drawbacks of poor solubility and many side effects of systemic administration of WA, improve the targeted accumulation at tumor sites, and enhance the inhibitory effect on neuroblastoma (97). As a natural biological vesicle has become an essential vehicle for treating many diseases, Li et al. designed and developed an engineered exosome with endogenously modified brain tumor targeting peptide and bound to magnetic nanoparticles by antibody complexation. A multifunctional nano drug (MNP@BQR@ANG-EXO-siGPX4) was subsequently constructed by loading small interfering RNA (siGPX4), a vital protein of the ferroptosis pathway GPX4, and Brequinar (BQR), an inhibitor of DHODH, onto the surface of exosomes and mesoporous silica, respectively (66). The nano drug can be enriched in the brain under local magnetic localization. The engineered exosomes modified with angiopep-2 (Ang) peptide can trigger transcytosis, allowing the particles to cross the BBB and target GBM cells by recognizing the LRP-1 receptor. The synergistic ferroptosis treatment of GBM is achieved by the triple action of catabolism of dihydrolactate dehydrogenase and glutathione peroxidase four ferritin defense axis, combined with Fe_3_O_4_ nanoparticle-mediated Fe^2+^ release. The results of this study suggest that this nano drug provides a new idea for enhanced ferroptosis for the synergistic treatment of GBM (66). Protein disulfide isomerase (PDI) has the hazard of interfering with nascent proteins to worsen glioma disease. PID is generally overexpressed in glioma cells, maintaining redox stability in tumor cells. Glioma cells also show a significant dependence on PID. Kyani et al. described a PDI nanomolecular inhibitor, 35GB, and showed that 35GB was able to upregulate the expression level of system Xc^-^ subunit SLC7A11 and inhibit the exchange function of system Xc^-^, resulting in decreased GSH synthesis and lipid peroxide pooling. 35GB also affects the expression of gene HMOX1, and overexpression of heme oxygenase-1, a source of supplied iron, disrupts iron metabolism in the organism and induces ferroptosis. 35GB can cross the blood-brain barrier and is expected to be a novel nano-inducer of ferroptosis in glioblastoma (98).

### 4.3 Other

Lipid peroxidation pooling is a significant feature of ferroptosis, and to induce ferroptosis in brain tumor cells *via* lipid metabolic pathways, free PUFAs must be activated (101). PUFAs are susceptible to oxidative free radical attack and lipid peroxidation. Supplementing PUFAs levels by exogenous or increasing intracellular levels of oxidized free radicals is extremely important for the induction of ferroptosis. There is a strong link between the level of PUFAs and ferroptosis in cancer cells, with the most vital ability to induce ferroptosis effect by linolenic acid in PUFAs (102). Zhou et al. developed an iron oxide particle (IO-LAHP) that replenishes PUFAs in the body by modifying linoleic acid hydroperoxides (LAHP) and hydrophilic oligomers on the surface of iron oxide nanoparticles (IO NPs) (99). Under acidic conditions, a Fenton-like reaction between Fe^2+^ ions released from iron oxide particles (IO-LAHP NPs) and linoleic acid hydroperoxides (LAHP) on the nanoparticle surface resulted in the formation of specific single-threaded oxygen (^1^O_2_) enabling tumor-specific therapy based on ROS-mediated mechanisms. *In vitro* cellular experiments demonstrated that IO-LAHP NPs could effectively increase intracellular ROS levels in glioma cells (U87MG), which induced ferroptosis in cancer cells. *In vivo* mouse tumor model experiments further confirmed their significant inhibitory effect on tumor growth. This also suggests that exogenous supplementation of *in vivo* PUFAs levels is feasible. The potential exists for this novel nanoparticle for the treatment of brain tumors. The relationship between lipid metabolism and ferroptosis is complex. There is not only an inducing relationship but also a possible inhibiting relationship, e.g., exogenous monounsaturated fatty acids (MUFAs), which can reduce the oxidative sensitivity of cells and inhibit the occurrence of ferroptosis (103). Therefore, the correct use of the relationship of lipid metabolism can have the most significant effect on eradicating brain tumor cells. Chen et al. used iron oxide nanoparticles loaded with paclitaxel to construct a nano drug (IONP@PTX). Using U251 and HMC3 as cell models, *in vitro* studies revealed that IONP@PTX inhibited cell migration and invasion ability, increased the levels of iron ions, ROS, and lipid peroxidation, enhanced the expression of autophagy-related proteins Beclin1 and LC3II, and inhibited the expression of p62 and ferroptosis-related protein GPX4 *in vitro* (104). *In vivo*, pharmacodynamic studies revealed that IONP@PTX significantly inhibited tumor volume in GBM xenografts and decreased the expression level of GPX4 protein in tumor tissues. Thus, IONP@PTX may inhibit GBM growth by enhancing the autophagy-dependent ferroptosis pathway and may be a potential ferroptosis inducer for ferroptosis-based tumor therapy (100).

## 5 Prospect and conclusion

Currently, the treatment and late convalescence of malignant brain tumors are clinically tricky, both to reduce the disruption of normal brain tissue cells during treatment and to protect essential central nervous functions of the brain. Ferroptosis, as a specific form of programmed cell death, is characterized by the accumulation of lipid peroxides. Its main mechanisms of occurrence and regulatory signals are still complex, involving the Fenton reaction, GPX4, and system Xc^-^, among others. Ferroptosis is essential in tumorigenesis and progression and is expected to be developed as a new cancer treatment strategy. The study of nano drug delivery systems has become a quality option in brain tumor treatment, overcoming some of the drawbacks of direct drug administration, prolonging the duration of drug action, and having great potential in improving drug efficacy. Intracellular iron is the basis of ferroptosis, and with the development of nanotechnology, various iron-based nanomaterials, such as iron oxide nanoparticles, amorphous iron nanoparticles, and organic iron frameworks, have shown attractive therapeutic advantages due to their ability to deliver exogenous iron to activate tumors (105). However, reliance on hydrogen peroxide for peroxide production is inefficient when brain tumor cells are under weakly acidic conditions, and excessive use of exogenous metals may cause potential adverse effects on human health, including acute and chronic damage (105, 106). Therefore, there is an urgent need to develop some non-iron-based nano drugs to induce ferroptosis.

Many studies have shown that combining multiple therapies is more advantageous than a single treatment strategy in clinical cancer treatment, such as efficient synergistic effects and reduced adverse effects with a single dose. Photodynamic therapy has been developed in the last century and licensed by the relevant regulatory authorities for cancer treatment. Similar to ferroptosis, they both produce ROS in cells. Photodynamic therapy has certain advantages in treating brain tumors. It can treat minimally invasive areas and protect other areas of the brain as much as possible, reducing the risk of treatment. By combining ferroptosis with photodynamic therapy, the anti-tumor effect is significantly enhanced. The tumor suppression rate is increased dramatically while reducing the possible adverse toxicity associated with ferroptosis treatment. The combination of ferroptosis with photodynamic therapy for combined anti-tumor therapy has been extensively studied, e.g., breast cancer, liver cancer, and lung cancer (106–108). However, there are fewer studies on brain tumors, which is a crucial direction for future research.

Tumor immunotherapy recognizes and kills tumor cells by stimulating the intrinsic immune system with minor damage to normal tissues. However, immunotherapy still has some problems in tumor treatment, such as immune response being only effective for a small proportion of patients and low efficiency due to insufficient immunogenicity. Immunotherapy combined with ferroptosis has emerged as a promising and effective combination of cancer treatment. In terms of the combination mechanism, there is a potential relationship between immunotherapy itself and ferroptosis, as interferon γ released from immunotherapy-activated CD8^+^ T cells down-regulates the expression levels of mRNA and protein of two subunits of system Xc^-^, SLC7A11 and SLC3A2, inhibiting tumor cells of cystine uptake, thus promoting lipid peroxidation and ferroptosis of tumor cells; ferroptosis of tumor cells occurs along with the release of immunogenic antigens that induce immunogenic cell death of tumor cells, thus contributing to the anti-tumor efficacy of immunotherapy (109–111). Zhang et al. designed a bionic magnetic vesicle with leukocyte membranes containing the transforming factor-β inhibitor Ti, membranes wrapped with Fe_3_O_4_ magnetic nanoparticles, and programmed cell death antibody 1 (Pa) immobilized on the surface. In tumor cells, Ti and Pa can create an immunogenic microenvironment that increases intracellular hydrogen peroxide levels and promotes the Fenton reaction causing ferroptosis (112). Nanotherapy combining ferroptosis with immunotherapy is still a promising therapeutic option for tumors. It is expected to be applied to the treatment of brain tumors through further studies in the future.

The ferroptosis-based nano drug delivery system has provided many opportunities to treat brain tumors. However, it is still not entirely in the mature stage, and the biological safety of nano drugs is still a concern. Most of the research experiments are conducted on animals, and the practical application in the clinic is yet to be developed. Future research is expected to break through the bottlenecks and grow truly efficient and non-toxic anti-tumor nanomedicines.

## Author contributions

Conceptualization: YY, PJ, and ZY. Writing—original draft preparation: PJ, HC, JG, YX, PW, and LX. Writing—review and editing: YY, PJ, HC, and ZY. Supervision: YY and ZY. Project administration: PJ. Funding acquisition: YY and ZY. All authors have read and agreed to the published version of the manuscript. All authors contributed to the article and approved the submitted version.
